# Supplementary material for: pH-Responsive Lipid Nanocapsules: A Promising Strategy for Improved Resistant Melanoma Cell Internalization
Source: Cancers (Basel). 2021 Apr 22;13(9):2028. doi: 10.3390/cancers13092028 (PMC8122844; doi:10.3390/cancers13092028)
Supplement: Supplementary file 1 [file cancers-13-02028-s001.zip › cancers-1161747-supplementary.pdf]

pH-Responsive Lipid Nanocapsules: A Promising Strategy for Improved Resistant Melanoma Cell Internalization

Vincent Pautu, Elise Lepeltier, Adélie Mellinger, Jérémie Riou, Antoine Debuigne, Christine Jerome, Nicolas Clere and Catherine Passirani

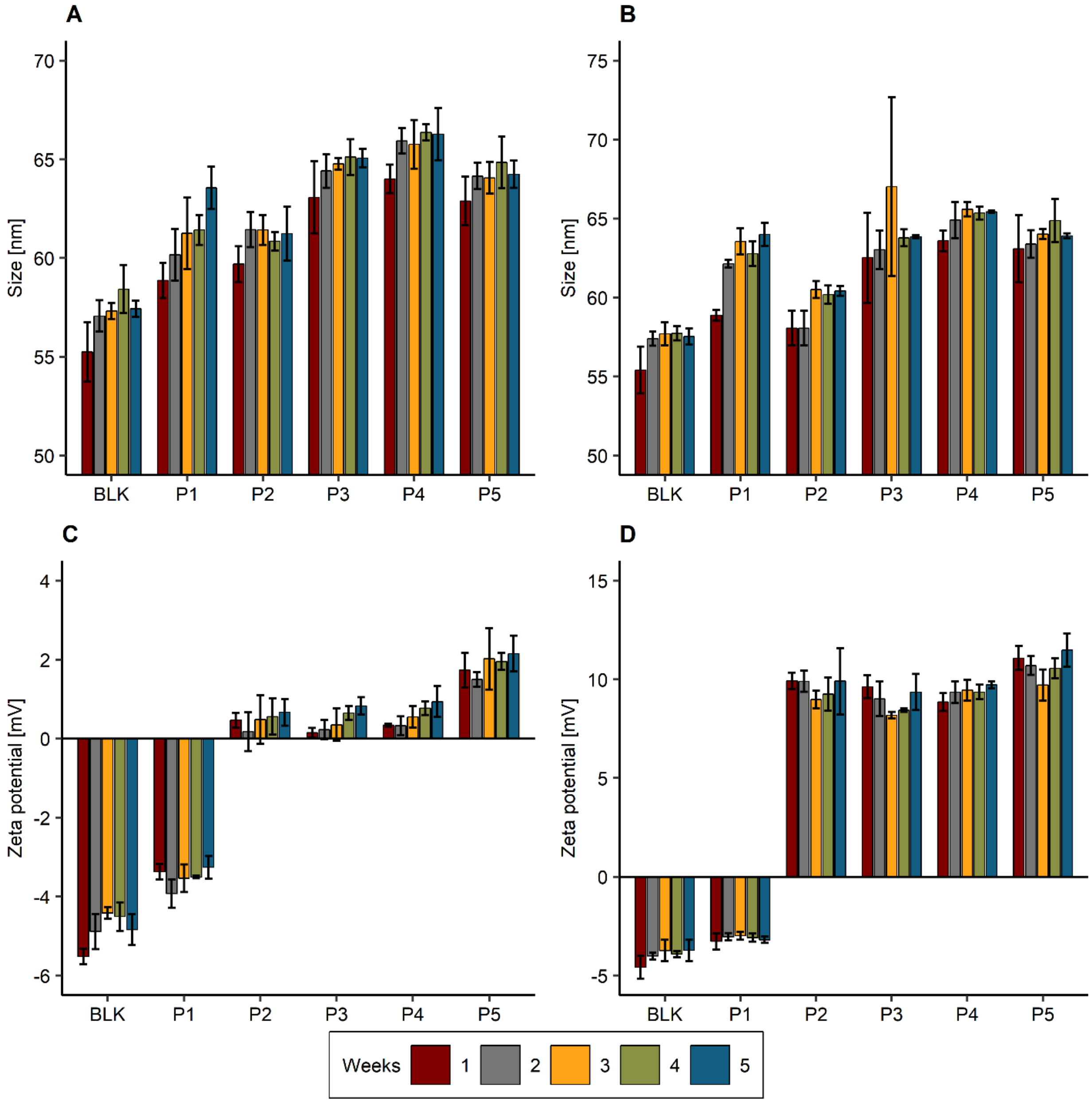

Figure S1. Stability of Blank LNC ("BLK") and modified LNC with polymer $C_{18}H_{37}$-$PNVP_{49}$ ("P1"), $C_{18}H_{37}$-P(NVP_{15}-co-Vim_5) ("P2"), $C_{18}H_{37}$-P(NVP_{22}-co-Vim_8) ("P3"), $C_{18}H_{37}$-P(NVP_{35}-co-Vim_{10}) ("P4") and $C_{18}H_{37}$-P(NVP_{21}-co-Vim_{15}) ("P5"). Stability was assessed by measuring the hydrodynamic diameter (nm) at pH 7.4 (A) and 6 (B), and zeta potential (mV) at pH 7.4 (C) and 6 (D) every week for 4 weeks. Results (n = 3) are expressed as mean measure ± standard deviation * $p < 0.05$.

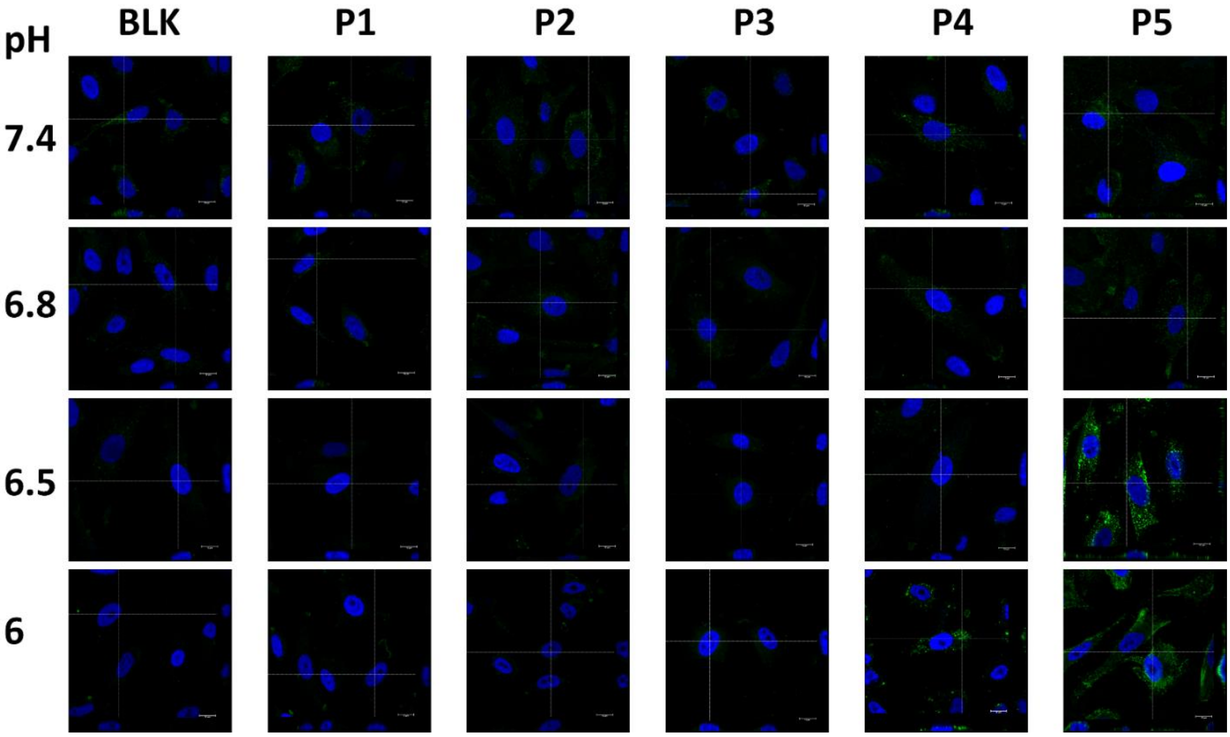

Figure S2. Confocal imaging of SK-Mel 28 cells after 2 h of incubation Blank LNC ("BLK") and LNC post-inserted by $C_{18}H_{37}$-$PNVP_{49}$ ("P1"), $C_{18}H_{37}$-P(NVP_{15}-co-Vim_5) ("P2"), $C_{18}H_{37}$-P(NVP_{22}-co-Vim_8) ("P3"), $C_{18}H_{37}$-P(NVP_{35}-co-Vim_{10}) ("P4") and $C_{18}H_{37}$-P(NVP_{21}-co-Vim_{15}) ("P5") at pH 7.4, 6.8, 6.5 and 6. Cell nucleus was stained with DAPI (in blue), green signal comes from the fluorescent LNC. Objective used: 63x/NA 1.40 oil with 2x numerical zoom, white lines represent two orthogonal sections used to analyze nanoparticle uptake. Scale bars correspond to 10 μm.
